# Maternal Obesity and MicroRNAs in Breast Milk: Implications for Infant Developmental Programming

**DOI:** 10.1111/obr.70098

**Published:** 2026-01-19

**Authors:** Gyslane M. Santos, Fabíola Isabel Suano de Souza, Luciana P. Pisani

**Affiliations:** ^1^ Department of Bioscience Federal University of São Paulo (UNIFESP) Santos São Paulo Brazil; ^2^ Department of Pediatrics Federal University of São Paulo São Paulo Brazil

**Keywords:** breastmilk, developmental programming, DOHaD, miRNA, obesity

## Abstract

This review explores the relationship between maternal obesity and alterations in the expression of microRNAs (miRNAs) in breast milk, highlighting how these changes may influence the developmental programming of the infant. Evidence suggests that maternal obesity can affect the bioactive composition of breast milk, including miRNA profiles, which are key regulators of metabolic and immune pathways in early life. Specific miRNAs, such as miR‐148a and miR‐30b, have been identified as modulators of metabolic processes, potentially impacting offspring growth, energy balance, and long‐term health outcomes. Additionally, maternal factors such as prepregnancy BMI and dietary patterns play a crucial role in shaping milk composition. Understanding these complex interactions is essential for informing nutritional strategies aimed at supporting optimal infant development and preventing chronic diseases later in life.

**Abbreviations**
AAP: American Academy of Pediatrics
Adrb3: adrenoceptor beta 3
AMPK: AMP‐activated protein kinase
BAT: brown adipose tissue
BMI: body mass index
DEXA: dual‐energy X‐ray absorptiometry
DNA: deoxyribonucleic acid
DOHaD: developmental origins of health and disease
EV: extracellular vesicle
GDM: gestational diabetes mellitus
GHD: gestational hypertension disorder
HOMA‐IR: homeostasis model assessment of insulin resistance
IADPSG: International Association of Diabetes and Pregnancy Study Groups
miRNA: microRNA
mRNA: messenger RNA
rWAT: retroperitoneal white adipose tissue
SAM: S‐adenosylmethionine
Ucp1: uncoupling protein 1
UPBEAT: Pregnancy Better Eating and Activity Trial
WD: western diet
WHO: World Health Organization

## 1 Introduction

Obesity and overweight are widely recognized as some of the greatest threats to global health in this century [1]. The most common definition is the one provided by the World Health Organization (WHO), marked by excessive or abnormal fat accumulation associated with adverse health outcomes [2, 3]. The magnitude of the problem is so great that obesity and overweight have the characteristics of a growing global epidemic. According to the WHO, there were more than 1 billion people in the world living with obesity in 2002, a number that could reach 2.3 billion in 2025 [2]. A more recent study suggests there will be about 3.8 billion (± 0.3b) adults living with obesity and overweight by 2050, if the growing trend continues [4], highlighting the vast magnitude of the problem for global public health. Maternal obesity is an increasingly prevalent condition, with significant implications for the health of both the mother and offspring on a transgenerational basis [5].

During pregnancy, obesity increases the risk of maternal complications including gestational diabetes, hypertension, premature birth, restricted intrauterine growth, and high birth weight. In children with obesity, there is an increased risk of malformations, respiratory and metabolic disorders in the neonatal period, and excess weight in childhood, adolescence, and adulthood [6]. Results from cohort studies with individuals born small for gestational age [7, 8], children of women exposed to famine during wartime [9, 10] supported the concept of the developmental origin of health and disease (DOHaD) [11]. According to this concept, environmental changes in the fetal and neonatal stages can result in developmental adaptations, triggering physiological and metabolic responses in the offspring, such as hypertension, dyslipidemia, obesity, type 2 diabetes, and metabolic syndrome [12].

Breast milk is the primary source of complete nutrition for newborns, offering a combination of essential nutrients, antibodies, and hormones that promote healthy development. Breastfeeding is globally recommended for infant nutrition, with recommendations to initiate breastfeeding within the first hour after birth and to breastfeed exclusively until 6 months of age [13]. Breastfeeding promotes the health of the infant and the nursing mother in the short and long term. It is associated, in a dose‐dependent manner, with a reduction in infant mortality, incidence, and severity of infectious diseases such as diarrhea and respiratory tract infections. In the long term, breastfeeding is associated with lower risk of obesity, allergic diseases, type 2 diabetes, and better indicators of neuropsychomotor development in the baby in later life [14].

Among the biological components present in breast milk, microRNAs (miRNAs) stand out because of their regulatory role in gene expression and programming of fetal development [6, 15]. The miRNAs are a class of small noncoding RNA molecules that play pivotal roles in various biological processes by regulating the translation of messenger RNA (mRNA) into proteins [16]. During lactation, the presence of certain miRNAs in breast milk can influence the immune and metabolic development of the offspring. Most of the miRNAs present in human milk are derived from mammary epithelial cells [17]. Circulating microRNAs can also be useful as biomarkers for identifying populations at risk of developing chronic noncommunicable diseases [18]. Obesity can modify the composition of breast milk, including miRNA expression. Understanding the possible mechanisms of the milk‐borne miRNAs is fundamental to understanding the probable repercussions on the programming of fetal development and the risk of developing metabolic diseases in offspring [5]. In view of the above, the present narrative review aims to investigate the relationship between maternal obesity and changes in the expression of microRNAs in breast milk, seeking to identify how these changes affect the developmental programming of the offspring. The review will synthesize current knowledge and offer new perspectives that can guide future experimental research and clinical practice.

## 2 Material and Methods

### 2.1 Eligibility and Inclusion Criteria

This study consists of a narrative review, based on consultations of the academic databases Pubmed, Web of Science, and Scopus from January to March 2025. As inclusion criteria, studies were selected that analyzed miRNA in breast milk in animals and humans associated with obesity, including preclinical, experimental, clinical, randomized clinical, and controlled studies published in the last 10 years in English. After removing duplicate articles and review articles, we assessed the titles and abstracts. Potentially relevant articles were read in full to determine final eligibility.

After inserting the inclusion and exclusion criteria, 12 articles were selected, shown in Tables 1 and 2.

**TABLE 1:** Summary of the articles included in this review, according to the criteria established for carrying out the literature review.

References	Methods/humans	miRNAs	Main results
[19]	Milk from healthy mothers *n* = 47 and *n* = 18 with pregestational BMI obesity.	miR‐30b‐5p, miR‐4454, miR‐494‐3p, miRNAs let‐7, miR‐575, miR‐630, miR‐642a 3p, and miR‐652‐5p	miRNA in extracellular vesicles (bEVs) in mothers with obesity were differentially expressed, including miR‐575, miR‐630, miR‐642a‐3p, and miR‐652‐5p. These miRNAs and their target genes have been associated with cancer, neurological diseases such as Parkinson's, Alzheimer's, and psychological disorders.
[20]	Cohort of 59 mothers, milk collected (in Months 1–3) from healthy lactating women of normal weight n: 38 and overweight/obesity n: 21, with child growth monitored up to 2 years of age.	miR‐30a, miR‐146b, miR‐let7b, miR‐148a, miR‐27a, miR‐27b, miR‐222, miR‐103, miR‐200b, miR‐17, miR‐let7c, miR‐let7a, and miR‐181a	miRNAs miR‐103, miR‐17, miR‐181a, miR‐222, miR‐let7‐c, and miR‐146b are differentiated at 2 months in the case of maternal obesity. Reduced levels of leptin and adiponectin in the milk of mothers with overweight/obesity.
[21]	Cohort of 60 mothers, with normal weight *n* = 30 and overweight/obesity *n* = 30 (in Months 1 and 3) of lactation.	miR‐148a, miR‐29a, miR‐29b, miR‐30b, miR‐let‐7a, and miR‐32	Reduction of miRNA‐148a in the group with overweight/obesity, this decrease may increase the risk of childhood obesity and negatively affect the neurological development of children born to mothers with obesity.
[22]	Prospective cohort, 364 nursing mothers, milk was collected between days (2 and 74) after birth. The milk was then frozen at −80°C.	Sequencing of 1523 miRNAs	The expression of miRNAs was associated with the time of milk collection and maternal BMI. The most expressed miRNAs (bEVs) in human milk were involved in pathways related to the endocrine system, cells, neurodevelopment, and cancers.
[23]	86 mothers, *n* = 33 mothers with pregestational obesity, collected colostrum samples on days (2–5 postnatal) and mature milk (3 months). The milk was subsequently frozen at −80°C. Anthropometric data and clinical conditions were collected.	miR‐Let‐7a, miR‐378, and miR‐30b	miRNA‐let‐7a, miRNA‐30b, and miRNA‐378 were expressed in colostrum and mature milk and altered with maternal pregestational BMI, gestational weight gain, and lactation period.
[24]	A longitudinal cohort of 192 mothers examined 503 samples of breast milk. The milk was collected in the months (0, 1, and 4) after birth. Sequencing quantified miRNAs within the lipid fraction.	Sequencing of 238 miRNAs	86% of miRNAs were affected by milk maturity; 54% increased from 0 to 4 months. Few miRNAs were affected by maternal age, race, parity, BMI and gestational diabetes. However, almost half of the abundant miRNAs were impacted by diet.
[25]	A cohort of 54 mothers were examined, blood and breast milk samples were taken, and milk was collected (6 weeks and in the second trimester) after birth.	Sequencing of 798 miRNAs	miRNA counts were lower among multiparous women with a pregestational BMI > 25 kg/m^2^.
[26]	The cohort examined 166 nursing mothers, whose milk was collected in Months 1 and 4 of lactation. It was stored at −20°C and then at −80°C. The weight of the offspring was collected from the medical records	Sequencing of 1755 miRNAs, selecting the miRNAs related to growth and weight gain, miR‐30a‐5p, miR‐141‐3p, miR‐374b‐5p, miR‐29a‐3p, miR‐224‐5p, miR‐200a‐3p, mi151a‐3p, miR103a‐3p, and let‐7i‐5p	The miRNA profile of breast milk was related to weight for length (WFL) and miR 224‐5p was associated with conditional weight gain (CWG) in the *Z* score at 12 months.

**TABLE 2:** Summary of the articles on preclinical studies in animal models included in this review, according to the criteria established for carrying out the literature review.

References	Method/Wistar rats	miRNA	Main results
[27]	Milk samples are collected from mother rats given DP control (*n* = 8), cafeteria (*n* = 8) and post‐cafeteria (*n* = 10) diets at three points during lactation (days 5, 10 and 15).	miR‐222, miR‐203, miR‐200a, miR‐103, miR‐27a, and miR‐26a	Levels of miR‐222 increase in milk, whereas miR‐103 and miR‐27 decrease during lactation. On the 15th day of lactation, cafeteria mothers showed higher levels of miR‐222 and lower levels of miR‐200a and miR‐26a than the control group. Cafeteria diet intake in lactating rats, and not obesity itself, leads to changes in specific miRNA levels.
[28]	Female rats, fed either a standard diet, DP control group (*n* = 8), or a diet high in fat and sucrose, WD Western diet (*n* = 9), for 1 month. Mothers in the control group continued on the PD, but those fed the WD continued on the WD diet or were exposed to the PD during this period (reversal group: Rev. group; *n* = 10). At 3 points during lactation (Days 5, 10, and 15), milk samples were collected.	miR‐26a, miR‐27a, miR‐30d, miR‐99b, miR‐125a, miR‐181a, miR‐200a, miR‐200b, miR‐320a, miR‐331, and miR‐484	Increased levels of miR‐26a, miR‐222, and miR‐484 in the mammary gland in the WD group and miR‐26a, miR‐125a and miR‐222 in milk compared to controls. The WD group also showed higher levels of miR‐125a than the Rev. group. The implementation of a healthy diet during lactation normalizes the expression levels of specific miRNAs in the mammary gland of mothers affected by diet‐induced obesity but not in the milk.
[29]	Rats, fed a control diet (*n* = 7) or a diet enriched with oleic acid, betaine and leucine TX group (*n* = 7) throughout lactation. Milk collected (days 5 and 15) of lactation.	miR‐26a, miR‐27a, miR‐29a, miR‐103, miR‐200a, miR‐200b, miR‐221, and miR‐222.	The TX diet reduced the levels *of miR‐27a, miR‐103, miR‐200a, and miR‐222 i*n milk. In TX offspring, higher body fat was observed early and maintained into adulthood, accompanied by higher HOMA‐IR than controls at 3 months of age. The body fat of the offspring in adulthood correlated positively with the leptin and adiponectin ratio in milk on Day 15 and negatively with the miRNAs modulated by the TX diet.
[30]	Rats, fed a standard diet, control group CD (*n* = 10), or a diet high in fat and sucrose, Western diet WD (*n* = 9), for 1 month, and during gestation and lactation. Milk collection (Days 5, 10, and 15).	Microarray analysis detected 84 specific miRNAs probes	The WD diet reduced the expression of 36 miRNAs in milk, such as miR‐let‐7i‐5p and miR‐29a, which are related to genes that act on adipose tissue and increased thermogenesis.

The search strategy was adapted to the databases using the same search terms, alternating the terms “microRNA” OR “microRNA” OR “miRNA” AND “milk” OR “breast milk” OR “human milk” AND “obesity.”

## 3 Results and Discussion

### 3.1 Molecular Mechanisms of miRNAs

Human milk miRNAs are mainly synthesized by mammary epithelial cells (lactocytes) and are distributed in different milk fractions [31]. Extracellular vesicles (EVs) are small membranous structures delimited by lipid bilayers, released by cells into the extracellular environment [32]. They function as vehicles for intercellular communication, transporting proteins, lipids, and nucleic acids [33]. MiRNAs are found encapsulated in exosomes, a type of nanometric EV (approximately 30–150 nm) [32], or directly associated with the fat structure of milk [31, 34]. Studies of simulated gastric and pancreatic digestion in vitro have shown that milk exosome miRNAs remain stable and intact after passing through the digestive process. After surviving digestion, they are absorbed by intestinal epithelial cells [35].

After ingestion, miRNA absorption is facilitated by the immaturity of the newborn's gastrointestinal tract, which has greater permeability [36, 37]. In addition, the binding of miRNAs to lipoproteins protects them from degradation and aids in their transport to intestinal cells, where they are absorbed by endocytosis. There is evidence suggesting the participation of specific proteins, such as SID‐1 protein (SIDT1), which may act as miRNA carriers across cell membranes [38]. This indicates that there are specific mechanisms that facilitate the passage of miRNAs across cell membranes [38]. Animal studies have demonstrated an increase in specific miRNAs in plasma after consumption of pig or cow milk [39], confirming that these molecules are absorbed and reach target tissues [40]. Once in the bloodstream, miRNAs exert systemic effects by modulating gene expression in various tissues. Within cells, miRNAs can bind to messenger RNA (mRNA) molecules and regulate gene expression, which can influence the genes that are activated or repressed, thus affecting various biological processes [36].

**FIGURE 1:**
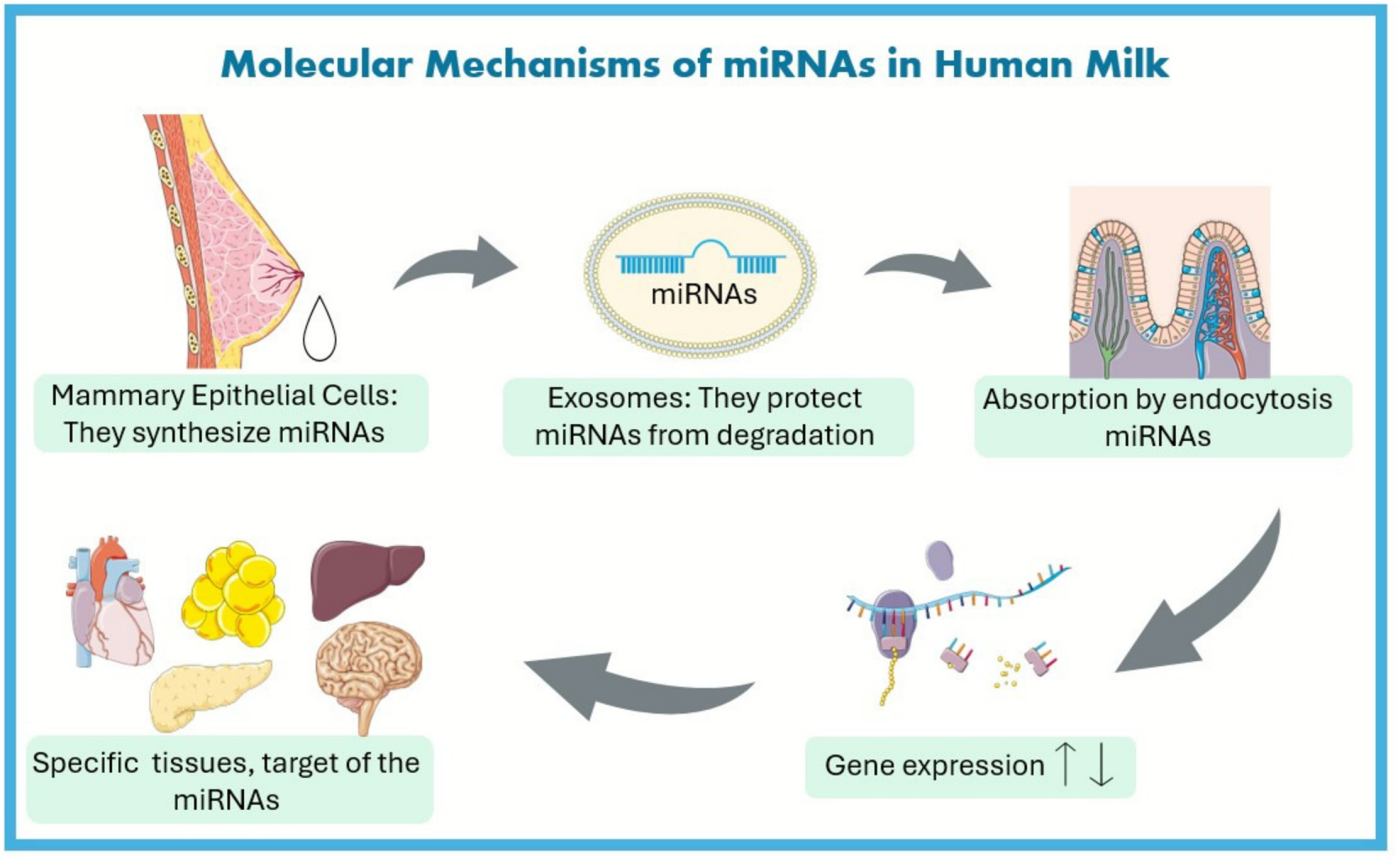
Molecular mechanisms of miRNAs in human milk. (1) The mammary glands synthesize miRNAs; (3) these miRNAs can also be encapsulated by exosomes, which protect against degradation; (3) absorption occurs through endocytosis; (4) the action of miRNAs on gene expression in target tissues (figure created using icons from smart.servier.com).

In human milk, variations in the amount of miRNAs identified can be attributed to several different factors, such as the populations studied, the stages of lactation analyzed, and the techniques used for detection and analysis [34, 41, 42]. Although the exact number may vary, human milk contains a wide and complex diversity of miRNAs [22, 41, 43]. These miRNAs play an important role in the development and health of neonates by acting on the immune system and neurocognitive and metabolic programming [44]. Figure 1 illustrates the mechanism of milk miRNAs.

### 3.2 The Influence of Obesity on the Composition of Breast Milk

Human milk is an essential food for children's health and development. The recommendations of the WHO and the American Academy of Pediatrics highlight the importance of exclusive breastfeeding in the first 6 months of life and as a complementary food for 2 years or more [45, 46, 47]. Its unique composition promotes healthy child development and reduces the risk of gastrointestinal and respiratory infections, thereby preventing allergies and childhood obesity [48]. Thus, exclusive breastfeeding can influence the health of the newborn directly and in the long term by preventing chronic diseases, such as type 2 diabetes mellitus and obesity [36, 44, 45, 49].

Maternal obesity, however, can alter this ideal composition, with significant implications for the baby's health. The UPBEAT (Pregnancy Better Eating and Activity Trial) was a clinical trial of over 1500 pregnant women with obesity in the United Kingdom. The trial demonstrated that the children born to women who underwent a lifestyle improvement intervention (diet, physical activity, and stress reduction) had reduced adiposity at 6 months of age. These positive outcomes were thought to be largely attributed to changes in breastfeeding practices and milk composition [15]. The mechanisms explaining this effect are not fully known, but studies show that maternal obesity influences the nutrient profile, levels of adipokines (leptin and adiponectin), and other bioactive molecules in breast milk, including miRNAs [5]. These molecules play a role in the baby's metabolic programming. Leptin, for example, is one of the main regulators of energy balance and has a strong relationship with maternal BMI [50].

Zamanillo et al. [20] observed that breast milk composition, including leptin and adiponectin concentrations, is related to maternal BMI. The variability in leptin supply is attributed to the main factor that conditions leptin secretion in milk: the content of maternal body fat reserves. In the first month of lactation, leptin levels were 2.8 times higher in the milk of mothers affected by overweight/obesity, whereas there was no significant difference in adiponectin levels between the groups. However, throughout lactation, leptin levels decreased in normal‐weight mothers but remained high in the group with overweight/obesity [20].

A prospective cohort study analyzed the breast milk of 365 mothers, and the expression of miRNAs—miR‐128‐3p, miR‐130a‐3p, miR‐574‐3p, and miR‐6881‐5p—was negatively correlated with maternal BMI. These miRNAs play an important role in the regulation of metabolic and inflammatory processes. miR‐128‐3p is associated with the inhibition of adipogenesis, whereas miR‐130a‐3p is also involved in the regulation of lipid metabolism and the production of prolactin, which is essential for lactation. miR‐574‐3p and miR‐6881‐5p act in the modulation of metabolic pathways that influence the processing of lipids and energy; this expression was decreased in mothers with high BMI; this change can be attributed to various conditions associated with excess weight, including insulin resistance, chronic inflammation, and hormonal changes [22].

Xi et al. [23] deepened this analysis of colostrum and mature milk throughout lactation, associated with maternal characteristics such as weight, lactation period, offspring sex, pregestational BMI, weight gain during gestation, and BMI at the end of gestation. The levels of miR let‐7a, miR‐30b, and miR‐378 in colostrum showed a negative relationship with maternal pregestational BMI, a result also found in the study by Kupsco et al. [22]. It was observed that miR‐30b and miR‐378 were positively correlated in the colostrum of mothers who had higher weight gain during pregnancy. Maternal BMI at the end of pregnancy was associated with lower miR‐let‐7a levels in mature milk. Another relevant finding was that miR‐378 and miR‐30b levels were higher in female infants. These miRNAs are related to metabolic processes and adipogenesis and may influence the development of adipose tissue and the regulation of body weight [23].

Studies of the composition of breast milk have revealed a complex relationship between miRNAs and infant development. Shah et al. [21] observed a reduction in miR‐148a and miR‐30b in the milk of mothers with overweight/obesity in the first month of lactation compared to the milk of normal‐weight mothers, 30% and 42%, respectively, but found no difference in 3‐month milk. The abundance of miR‐148a was negatively associated with infant weight, fat mass, and fat‐free mass during the first month of life. miR‐148a regulates genes related to energy metabolism and insulin signaling. In contrast, miR‐30b stimulates adipogenesis and positively regulates genes that promote fat storage [21].

In addition, Zamanillo et al. [20] showed that in mothers with a BMI < 25, there was an increase in the expression of miR‐30a, whereas in mothers affected by obesity, this expression was decreased, indicating that maternal BMI may affect the amount or regulation of this miRNA in milk. In addition, the expression of miR‐146b decreased in both groups throughout lactation. The six miRNAs that showed changes in expression profiles, miR‐let7a, miR‐103, miR‐222, miR‐17, miR‐146b, and miR‐30a, related to maternal BMI, are involved in biological processes relevant to child development and in important signaling pathways such as morphogenesis, cell differentiation, neurocognitive development, and the organization of lipid particles [51]. The composition of milk is influenced by maternal metabolic health. A decrease in the expression of miRNAs that regulate lipid metabolism and energy homeostasis can lead to dysregulation of these processes in the offspring, which can affect the metabolic health of infants [52, 53]. Variation in these miRNAs can have an impact on the growth and development of newborns, especially in the case of maternal obesity.

**FIGURE 2:**
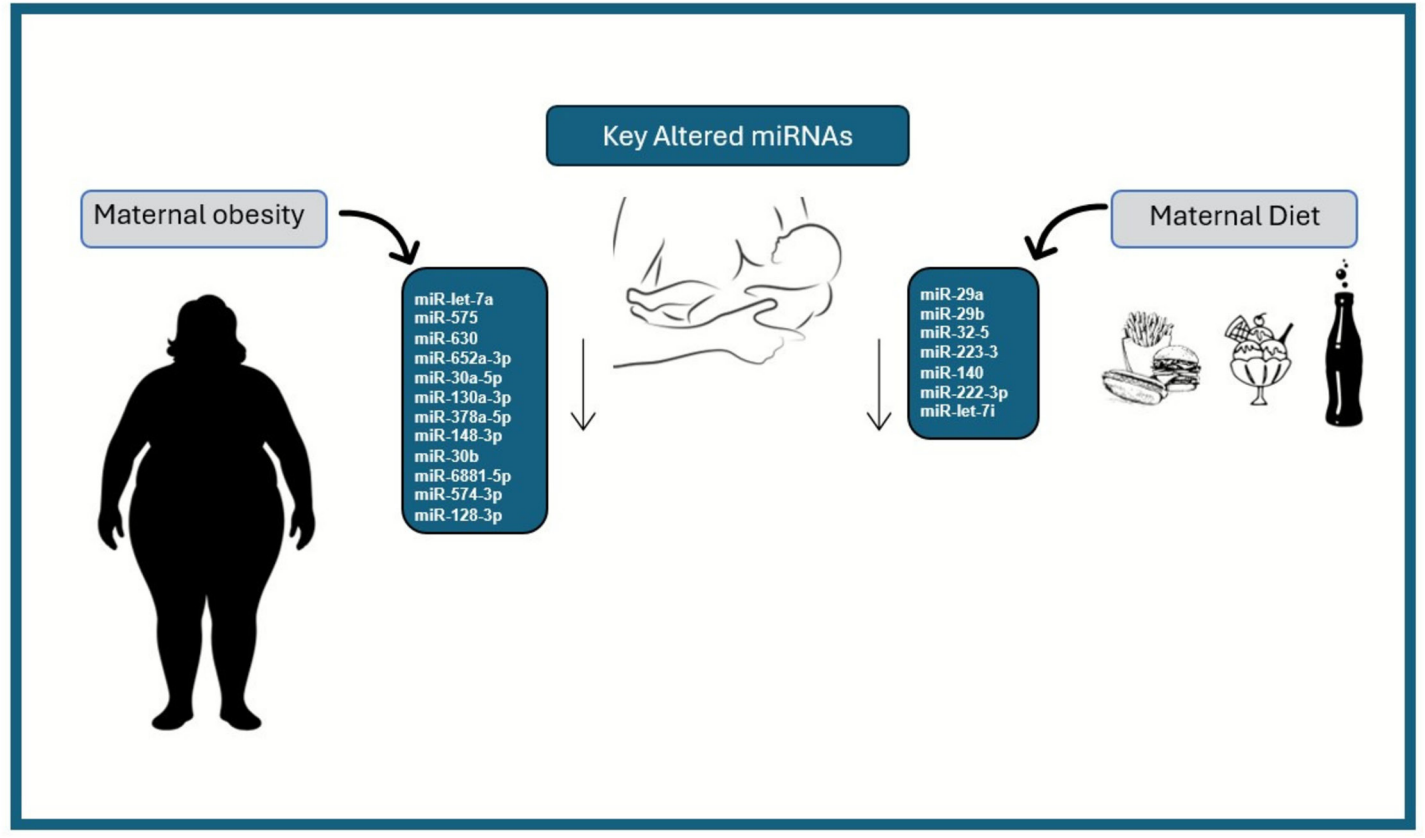
Changes in the expression of miRNAs due to maternal diet and maternal BMI.

Finally, a negative correlation was observed in the expression of miRNAs—miR‐103, miR‐17, miR‐let7‐c, miR‐222, miR‐181a, and miR‐146b—in normal‐weight mothers, indicating that the expression of these miRNAs was associated with a lower BMI in infants at 24 months [20]. The changes in breast milk miRNA expression associated with maternal BMI raise the hypothesis that these miRNAs may play a protective role against excessive weight gain in childhood and that breast milk may influence healthy growth [20, 21]. Figure 2 summarizes the changes in maternal diet and maternal BMI, highlighting how these variables influence the expression of miRNAs in breast milk.

### 3.3 Effects of Maternal Diet on Milk Composition and miRNAs

Perinatal nutrition, especially during lactation, is an important factor in neonatal programming. Maternal health plays a lasting role in infant development and has consequences that extend into adulthood [54]. Several studies explore how obesity and maternal diet impact the long‐term health of offspring, potentially predisposing individuals to chronic diseases such as obesity, diabetes, and cardiovascular disease [55, 56, 57, 58]. Epigenetic mechanisms have been proposed as a key event in metabolic programming [54, 56, 58]. During the postnatal period, the composition of breast milk and the mother's metabolic status can significantly influence the child's development and predispose them to metabolic outcomes later in life, such as insulin resistance, obesity, and metabolic syndrome. Changes in milk quality due to maternal diet or metabolic condition can affect the maturation of the central nervous system and other physiological pathways in offspring [59].

Research on how maternal diet affects the production of exosomes in milk is still scarce but is being explored. A notable study by Alonso‐Bernáldez et al. [29] investigated the effects of supplementation with betaine, leucine, and oleic acid in the diet of lactating Wistar rats, with the aim of evaluating the concentration of leptin and adiponectin, and miRNAs in breast milk, as well as the impact of these factors on mothers and offspring. The results showed that this diet increased the concentration of leptin and adiponectin in breast milk and modulation of the expression of miR‐27a, miR‐103, miR‐200a, and miR‐222 in milk on Day 15 of lactation, which correlates negatively with fat mass and changes in the HOMA‐IR index, indicating insulin resistance in offspring at 90 days of age. The altered miRNAs are related to leptin and adiponectin signaling, suggesting that they may be sensitive parameters for early postnatal programming of offspring [29]. Leptin and adiponectin are peptide hormones (adipokines) produced mainly by adipose tissue. Present in breast milk, they are considered bioactive factors that contribute to the metabolic programming of offspring. Their intake through milk can influence the development of the baby's neuroendocrine and metabolic systems [60, 61], which explains why the study by Alonso‐Bernáldez [29] included them as key variables.

Another study investigated the reduction in the expression of 36 miRNAs in the milk of rats fed a western diet (WD) before and during pregnancy or a control diet. Betaine was added to the western diet during lactation. Maternal exposure to WD negatively affected the expression of miRNAs in milk; among the most affected miRNAs were miR‐222‐3p, miR‐32‐5p, miR‐140‐5p, miR‐29c‐3p, miR‐29a‐3p, and let‐7i‐5p. In addition, the WD diet was associated with increased adiposity in offspring, showing a difference between males and females. The authors explored the potential effects of these changes on offspring health by evaluating the expression of target genes in retroperitoneal adipose tissue (rWAT) and brown adipose tissue (BAT), focusing on the regulation of lipid metabolism. The reduction of let‐7i‐5p and miR‐29a was associated with increased expression of genes such as Ucp1 and Adrb3, suggesting that betaine supplementation may induce a greater thermogenic capacity in females [30].

**FIGURE 3:**
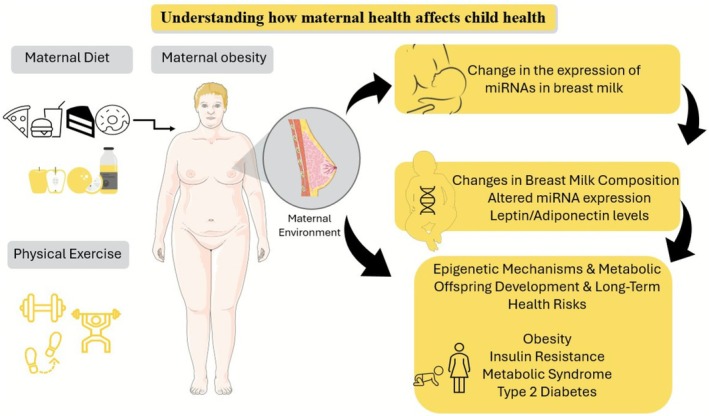
Schematic representation of factors related to maternal lifestyle significantly influence the development of obesity, which in turn can alter breast milk composition, including the expression of microRNAs (miRNAs). These changes may affect gene expression in the infant, contributing to developmental programming and increasing the risk of metabolic diseases later in life. It is important to note that the findings summarized in this figure are predominantly based on studies conducted in animal models.

The effect of obesogenic diets during lactation in rats showed that the maternal diet has an impact on the concentration of miRNAs in milk. The miRNAs miR‐222, miR‐203, miR‐103, miR‐27a, miR‐200a, and miR‐26a were analyzed, and the results indicated that mothers who received an obesogenic diet showed changes in the concentrations of these miRNAs compared to mothers who were on a control diet [27]. On the other hand, the implementation of a healthy diet during lactation in rats with obesity induced by an obesogenic diet attenuated the harmful effects of metabolic programming in the offspring. This nutritional intervention promoted the normalization of the concentration of specific miRNAs (miR‐26a, miR‐222, and miR‐484) in the mammary glands of mothers. However, this normalization was not observed in the levels of these miRNAs in breast milk, which suggests that the composition of the milk may not have been affected by changes in the mother's diet [28]. The normalized miRNAs miR‐26a and miR‐222 are associated with the regulation of the cell cycle and the stress response and may play a role in oncogenesis, whereas miR‐484 is related to the regulation of genes involved in cell development and function, including metabolic processes [62, 63, 64]. Although there are studies suggesting a possible influence, there is still no comprehensive understanding of how the mother's diet affects the expression of miRNAs in milk. It is essential to advance research to determine direct cause‐and‐effect relationships and their precise impact on infant health. Based primarily on evidence from animal models, Figure 3 illustrates how maternal diet and obesity influence the expression of miRNAs in breast milk, which in turn can cause modifications in gene expression and impact child development.

### 3.4 Dynamics of miRNA Expression Throughout Lactation

The influence of maternal miRNAs on the offspring extends beyond the first few weeks of life. For example, a study by van Syoc et al. [26] investigated the link between milk miRNAs and childhood obesity during the first year of life; the weight status of the neonate at different ages (1 and 4 months) showed no significant differences in miRNA profiles. However, they observed a relationship between miR‐224‐5p in breast milk and the weight growth status of infants at 12 months. miR‐224‐5p had a predictive association with childhood obesity status but was not related to maternal pregestational weight. The function of this miRNA is related to adipocyte differentiation and fatty acid metabolism, although its exact role and the extent of its influence still require further investigation [26]. In addition to the influence of specific miRNAs, the composition of breast milk is affected by maternal characteristics. Several studies indicate that the mother's body mass index (BMI) is a determining factor; Muse et al. [25] conducted a pilot study involving 54 mother–child dyads on the total miRNA count in EVs in prenatal plasma during the second trimester of pregnancy and in breast milk after 6 weeks of lactation, with the aim of identifying factors that influence their composition and discussing their role in mother–child communication. The findings showed that the count was lower in the milk of mothers with a BMI greater than 25 kg/m^2^, compared to mothers with a BMI of > 25 kg/m^2^. Other factors such as maternal age, gestational weight gain, baby sex, and gestational age at delivery were not significantly associated with the miRNA composition of milk [25]. Cho et al. [19] analyzed 798 miRNAs and found significantly lower counts of miRNA EVs in the milk of mothers with a BMI ≥ 30. This differentiation occurred in 19 miRNAs. The identified included miR‐575, miR‐630, miR‐642a‐3p, and miR‐652‐5p. The researchers linked the altered expression of these miRNAs to neurological and psychological disorders through their predicted target genes and associated pathways. TP53, a gene that encodes the tumor suppressor protein p53, was a central gene within this network and was predicted to be activated. The dysregulation of TP53 has been linked to various neurological and psychological conditions. This research suggests that maternal obesity may influence infant neurological development through the altered miRNA content of breast milk [19]. These findings suggest that the BMI of the mothers before pregnancy may interfere with the composition of breast milk [25]. This reduction can be attributed to the metabolic and inflammatory factors often associated with overweight and obesity, which can influence the production and release of miRNAs by mammary cells [59].

The dynamics of miRNA expression are also influenced by other factors throughout lactation. Hicks et al. [24] investigated breast milk samples from 192 mothers and found that milk maturation significantly influences miRNA levels, with a marked increase during the first 4 months postpartum. Additionally, only a few maternal characteristics, such as age, race, diet, and BMI, were found to affect miRNA expression. Notably, four miRNAs were associated with maternal dairy intake. These findings underscore the important role of maternal diet in shaping miRNA profiles, suggesting that targeted nutritional interventions could support infant metabolic and immune health [24].

Studies on the relationship between maternal obesity and microRNAs (miRNAs) in breast milk face significant challenges, particularly in relation to sample size. Cho et al. [19] note that many previous studies analyzed only a few miRNAs, which limited a complete understanding of their potential impacts. In contrast, more recent studies, such as those by Hicks et al. (*N* = 192) and Kupsco et al. (*N* = 364), have larger samples, providing more robust data. However, even in one of the largest studies (van Syoc et al., *N* = 163), the low incidence of childhood obesity in the cohort (< 10%) limited the ability to detect statistically significant differences, highlighting the need for further research with larger samples, different milk fractions, and more diverse populations.

### 3.5 Therapeutic Potential and Future Directions

MiRNAs, due to their ability to regulate multiple genes and metabolic pathways [65], represent promising targets for nanomedicine, especially in therapeutic interventions targeting specific diseases [66, 67]. Targeted delivery of miRNAs can modulate biological and pathological processes, such as cell differentiation and regulation of immune responses. In the field of neonatology, exosomes have shown great potential in the treatment of necrotizing enterocolitis (NEC). A recent review [68] elucidated the important role of breast milk exosomes in the intestinal health of newborns, especially those at high risk due to their ability to modulate cell differentiation, immune signaling, and intestinal integrity [68].

Preclinical studies have validated this potential in various therapeutic contexts. MiR‐148a‐3p, present in human breast milk exosomes, has been shown to protect against necrotizing enterocolitis (NEC) in mouse models by regulating p53 and SIRT1 proteins to reduce intestinal inflammation. Similarly, a synthetic agomir of miR‐148a‐3p showed similar efficacy, indicating a potential therapeutic strategy for NEC [69]. In a different context, goat milk exosomes have shown potent antiviral activity against dengue virus (DENV), paving the way for the use of exosomes as therapeutic agents against viral diseases such as DENV and ZIKV [70].

### 3.6 Challenges and Limitations in miRNA Therapy

Despite its potential, miRNA therapy faces significant challenges that need to be overcome. The efficacy of miRNAs depends on their bioavailability and stability in the body, which requires the development of robust delivery vehicles, such as exosomes [40]. In addition, the complexity and costs of purifying exosomes from natural sources, such as milk, are important limitations, encouraging research into synthetic analogues. Other concerns include delivery specificity and the risk of off‐target effects, as well as the immune responses that may be triggered by these treatments [36, 51].

### 3.7 Next Steps and Future Directions for Research

For miRNA‐based therapies to advance, further mechanistic studies are needed to elucidate the specific role of miRNAs in different diseases. The main future direction involves the transition from preclinical models to validation in clinical studies. Research should also focus on developing new technologies for targeted miRNA delivery and exploring combinations of miRNAs, which may offer a more effective and comprehensive approach to treating complex diseases. In addition to their therapeutic potential, EV miRNAs also stand out as biomarkers for the diagnosis and prognosis of diseases such as breast cancer and type 2 diabetes [71].

## 4 Conclusion

Emerging evidence suggests that maternal obesity reshapes the composition of breast milk, specifically with regard to microRNA (miRNA) profiles. Changes observed in miRNAs such as miR‐148a and miR‐30b, which are directly linked to metabolic pathways, suggest a mechanism by which early metabolic programming in children may be altered. The negative correlation between the expression of certain miRNAs and the infant's BMI offers a direct link, indicating that the bioactivity of breast milk may influence growth and fat accumulation in childhood. Although the current body of evidence is promising, we recognize that longitudinal and mechanistic studies directly demonstrating the effects of altered miRNA expression in breast milk on long‐term health outcomes in children are still lacking. Therefore, the identification of these miRNAs not only reinforces the importance of breast milk as a health programming factor but also highlights the urgency of future research. The critical next step is to conduct robust translational studies to validate these hypotheses, allowing for a complete understanding of how maternal nutrition and subsequent changes in breast milk contribute to metabolic health and disease in offspring, solidifying the role of early nutritional intervention as a preventive strategy.

## Funding

This work was supported by the Conselho Nacional de Desenvolvimento Científico e Tecnológico (307305/2023‐6).

## Conflicts of Interest

The authors declare no conflicts of interest.

## Data Availability

Data sharing is not applicable to this article, as no datasets were generated or analyzed during the current study.
